# Differential associations of fetuin-A and calcification propensity with cardiovascular events and subsequent mortality in patients undergoing hemodialysis

**DOI:** 10.1093/ckj/sfae042

**Published:** 2024-02-29

**Authors:** Katsuhito Mori, Tetsuo Shoji, Shinya Nakatani, Hideki Uedono, Akinobu Ochi, Hisako Yoshida, Yasuo Imanishi, Tomoaki Morioka, Yoshihiro Tsujimoto, Makoto Kuro-o, Masanori Emoto

**Affiliations:** Department of Nephrology, Osaka Metropolitan University Graduate School of Medicine, Osaka, Japan; Department of Vascular Medicine, Osaka Metropolitan University Graduate School of Medicine, Osaka, Japan; Vascular Science Center for Translational Research, Osaka Metropolitan University Graduate School of Medicine, Osaka, Japan; Department of Metabolism, Endocrinology and Molecular Medicine, Osaka Metropolitan University Graduate School of Medicine, Osaka, Japan; Department of Metabolism, Endocrinology and Molecular Medicine, Osaka Metropolitan University Graduate School of Medicine, Osaka, Japan; Department of Metabolism, Endocrinology and Molecular Medicine, Osaka Metropolitan University Graduate School of Medicine, Osaka, Japan; Department of Medical Statistics, Osaka Metropolitan University Graduate School of Medicine, Osaka, Japan; Department of Metabolism, Endocrinology and Molecular Medicine, Osaka Metropolitan University Graduate School of Medicine, Osaka, Japan; Department of Metabolism, Endocrinology and Molecular Medicine, Osaka Metropolitan University Graduate School of Medicine, Osaka, Japan; Division of Internal Medicine, Inoue Hospital, Suita, Osaka, Japan; Center for Molecular Medicine, Jichi Medical University, Shimotsuke, Tochigi, Japan; Department of Nephrology, Osaka Metropolitan University Graduate School of Medicine, Osaka, Japan; Department of Vascular Medicine, Osaka Metropolitan University Graduate School of Medicine, Osaka, Japan; Department of Metabolism, Endocrinology and Molecular Medicine, Osaka Metropolitan University Graduate School of Medicine, Osaka, Japan

**Keywords:** calcification propensity, cardiovascular disease (CVD), fetuin-A, hemodialysis, T50

## Abstract

**Background:**

Fetuin-A inhibits precipitation of calcium-phosphate crystals by forming calciprotein particles (CPP). A novel T50 test, which measures transformation time from primary to secondary CPP, is an index for calcification propensity. Both lower fetuin-A and shorter T50 levels were associated with cardiovascular disease (CVD) risk in patients with chronic kidney disease (CKD). Extremely high risk for CVD death in advanced CKD patients consists of high-incidental CVD event and high mortality after CVD event. To date, it is unclear whether fetuin-A and/or T50 can equally predict each CVD outcome.

**Methods:**

This prospective cohort study examined patients undergoing maintenance hemodialysis. The exposures were fetuin-A and T50. The outcomes of interests were new CVD events and subsequent deaths. The patients were categorized into tertiles of fetuin-A or T50 (T1 to T3).

**Results:**

We identified 190 new CVD events during the 5-year follow-up of the 513 patients and 59 deaths subsequent to the CVD events during 2.5-year follow-up. A lower fetuin-A but not T50 was significantly associated with new CVD events [subdistribution hazard ratio (HR) 1.73, 95% confidence interval (CI) 1.15–2.61, *P* = .009 for T1 vs T3]. In contrast, a shorter T50 but not fetuin-A was a significant predictor of deaths after CVD events (HR 3.31, 95% CI 1.42–7.74, *P* = .006 for T1 + T2 vs T3). A lower fetuin-A was predictive of new CVD events, whereas a shorter T50 was more preferentially associated with subsequent death.

**Conclusion:**

These results indicate that fetuin-A and T50 are involved in cardiovascular risk in different manners.

## Graphical Abstract

**Graphical Abstract:**
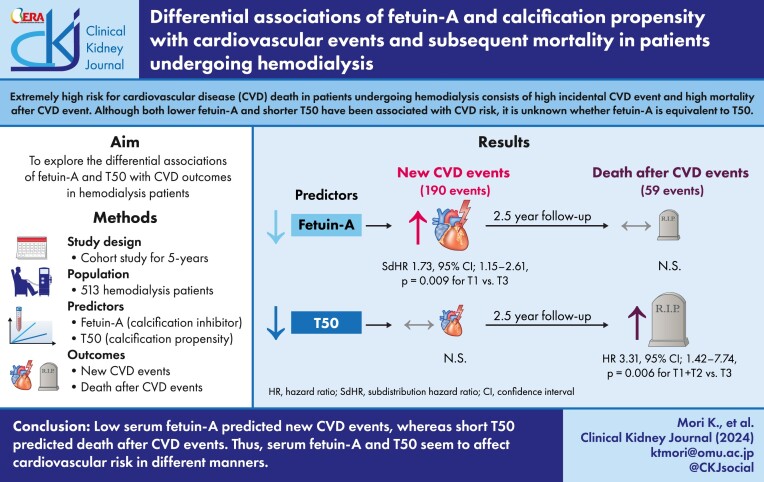

KEY LEARNING POINTS **What was known:**

- Patients undergoing hemodialysis have an extremely high risk of cardiovascular disease (CVD) death, which consists of both high incidence of CVD events and high mortality rates after CVD events.
- Lower fetuin-A, an inhibitor of vascular calcification, and shorter T50, an index for calcification propensity, have been associated with CVD risk.

**This study adds:**

- We found that lower fetuin-A, but not T50, was significantly associated with new CVD events.
- On the other hand, shorter T50, but not fetuin-A, was a significant predictor of deaths after CVD events. It appears that fetuin-A and T50 are involved in CVD risk in different manners in hemodialysis patients.

**Potential impact:**

- Careful monitoring of both fetuin-A and T50 may lead to improvements in CVD outcomes.

## INTRODUCTION

Fetuin-A (α2-Heremans Schmid glycoprotein: AHSG) is known to be a predictor of cardiovascular disease (CVD) and all-cause mortality in patients undergoing dialysis who are prone to vascular calcification [1–3]. *In vitro* and *in vivo* findings have suggested that fetuin-A can prevent ectopic calcification by forming colloidal mineral complexes, termed calciprotein particles (CPP), which consist of mainly calcium, phosphate and fetuin-A [4–8]. Since fetuin-A can buffer super saturation of mineral ions, the association of lower fetuin-A and worse clinical outcomes can be explained at least partly by lower protection against vascular calcification. In addition, fetuin-A may affect cardiovascular outcomes through its effect on insulin resistance [8–10].

In the early phase of the calcification process, primary CPP are formed to prevent precipitation of calcium-phosphate crystals. Further calcium and phosphate load accelerate conversion of primary CPP into larger secondary CPP which contain hydroxyapatite crystals [4–7]. Recently, a novel serum test “T50” has been developed, which is the transformation time from primary to secondary CPP *in vitro* measured by detecting rapid increase of turbidity by nephelometric method [11]. Although fetuin-A is a dominant contributor of T50, phosphate, calcium, magnesium and albumin levels are also known to influence T50. Thus, T50 is an integrated indicator of various factors of chronic kidney disease (CKD)–mineral and bone disorder (MBD).

A shorter T50, which means higher calcification propensity, was associated with higher risk for mortality, CVD events and kidney failure in patients with CKD stage 2–4 [12, 13]. An inverse association between T50 and all-cause or CVD death was found in patients undergoing hemodialysis [14, 15]. In kidney transplant recipients, a shorter T50 was a potent predictor of CVD events [16], as well as all-cause and CVD-related mortality [17, 18].

Patients undergoing hemodialysis suffer from an extremely increased CVD mortality compared with the general population [19]. The patients have higher risk not only for incidence of a CVD event but also for subsequent death after a CVD event [20, 21]. Thus, these two components, i.e. CVD events and subsequent death, synergistically raise the CVD mortality [19]. It has been reported that the factors affecting death after CVD events were different from those affecting new CVD events in patients undergoing hemodialysis [22–24].

Although the previous studies suggested a considerable overlap between fetuin-A and T50 as predictor of CVD events and mortality, it is unknown whether fetuin-A is equivalent to T50. In the present study, we compared fetuin-A and T50 in terms of the associations with new CVD events and death after CVD events in a cohort of hemodialysis patients.

## MATERIALS AND METHODS

### Study design and participants

This study was performed using data of the ‘DREAM’ (Dialysis-Related Endocrine and Metabolic changes affecting cardiovascular disease) cohort, which is a prospective, observational, and single-center study as previously registered at the UMIN-CTR (unique ID: UMIN000006168, http://www.umin.ac.jp/ctr/index.htm). Some of the results of this cohort have been reported previously [24, 25]. The DREAM cohort was composed of total 518 patients undergoing hemodialysis at Inoue Hospital, Suita, Osaka, Japan. The ethics committee at Inoue Hospital approved the study protocol (Approval No. 121), which adhered to the Declaration of Helsinki. All participants provided written informed consent and were enrolled in December 2004 and followed up until December 2009. Of the 518 patients, 513 patients with available data on both fetuin-A and T50 were analyzed in this study.

### Blood sampling and laboratory tests

Blood samples for routine laboratory tests were taken before initiation of hemodialysis session on the first day of the week (Monday or Tuesday). Serum samples at baseline were frozen and kept at −80°C until the time of additional assays.

### Serum fetuin-A assay

Serum fetuin-A was measured by a commercially available enzyme-linked immunosorbent assay kit (BioVender Laboratory Medicine, Inc., Czech Republic) as previously reported [9, 26].

### Measurement of serum calcification propensity (T50)

Serum calcification propensity (T50) was quantified according to the method originally developed by Pasch *et al*. [11]. In brief, serum samples were supersaturated by adding calcium (final concentration of 10 mM) and phosphate (final concentration of 6 mM) to trigger formation of primary CPP which mainly contains fetuin-A and amorphous calcium phosphate. Primary CPP undergoes spontaneous transformation into secondary CPP which includes thermodynamically stable hydroxyapatite crystals. To detect the transition from primary to secondary CPP, time to a rapid increase in turbidity during a thermo-constant incubation at 37°C was quantified by a time-resolved laser light scatter nephelometry using an automated laser-based microplate nephelometer (Nephelostar; BMG Labteck, Offenberg, Germany). The one-half transition time (T50) was determined as calcification propensity specific for individual serum. Thus, a shorter T50 means lower resistance to crystallization and higher calcification propensity. All serum samples were measured in duplicate, and the average values were used for analysis. The CV of inter- and intra-assay were 3.20% and 4.47%, respectively, in our laboratory [27, 28].

### Definition of CVD events

CVD events in the DREAM cohort were defined as the new occurrence of ischemic heart disease, ischemic stroke, hemorrhagic stroke, peripheral artery disease, pulmonary edema and cardiac valve disease as previously described [24, 25]. Incident and recurrent CVD and interventions for CVD were included as CVD events whereas chest pain or transient ischemic attack with no evidence with images were excluded. The same definition was applied to the pre-existing CVD at baseline and the new onset of CVD during the follow-up. Sudden deaths were considered as new and fatal CVD events. Furthermore, new CVD events were divided into atherosclerotic CVD events (ischemic heart disease, ischemic stroke and peripheral artery disease) and non-atherosclerotic CVD events (hemorrhagic stroke, pulmonary edema and cardiac valve disease). The patients with sudden death were classified into atherosclerotic CVD events if they had history of CVD events.

### Study outcomes

The key outcomes of interest were new CVD events and deaths after CVD events in this study. All-cause mortality was also evaluated as an additional outcome to confirm the previously reports [1, 2, 14, 15].

### Other variables

As the major demographic factors, we documented age, sex, duration of hemodialysis, clinical diagnosis of diabetic kidney disease (DKD) (not necessarily biopsy proven) and pre-existing CVD. Serum calcium, phosphate, intact parathyroid hormone (PTH) and use of vitamin D receptor activator (VDRA) were recorded as the parameters of CKD-MBD. Calcium-sensing receptor agonists were not used at baseline in this study. As clinical factors of anemia in CKD, we included hematocrit, dose of erythropoiesis-stimulating agent (ESA) and use of intravenous iron injections. At baseline, only recombinant human erythropoietin preparations were available as ESA in Japan. We considered hypertension, current smoking status, and dyslipidemia as traditional risk factors. We defined hypertension as blood pressure of 140/90 mmHg or higher and/or use of any anti-hypertensive medication according to the guideline of Japanese Society of Hypertension [29]. Dyslipidemia was defined as high-density lipoprotein cholesterol (HDL-C) ≤40 mg/dL, non-HDL-C ≥150 mg/dL and/or use of statin, with these lipid levels being derived from the target levels for patients with CKD recommended by the guideline of Japan Atherosclerosis Society [30]. As indicators of inflammation and wasting, body mass index after dialysis, C-reactive protein (CRP) and serum albumin were recorded.

### Follow-up and clinical endpoints

The cohort was followed-up for up to 5 years until the end of 2009. At the end of each year, primary physicians made up an annual follow-up sheet to report dates of death (cause of death), CVD events, kidney transplantation, switching to peritoneal dialysis and moving away to other dialysis unit, as previously reported [24, 25].

### Statistics

The patients were divided into tertiles of fetuin-A and T50, and their characteristics were summarized as numbers, percentages or medians (interquartile ranges) as indicated. Comparisons in prevalence and median values among the tertiles were performed by χ^2^ test and Kruskal–Wallis test, respectively. Unadjusted correlations of fetuin-A with other variables were examined by Spearman's rank correlation. CRP values below detection limit (0.01 mg/dL) were handled as 0.01 mg/dL.

Cumulative incidence of new CVD events was evaluated by Gray's test according to the tertiles of fetuin-A and T50, because death would impede the occurrence of CVD events [31, 32]. The associations of fetuin-A and T50 with new CVD events were examined by multivariable-adjusted Fine–Gray models which consider the competitive risk of death. Subdistribution hazard ratios (sdHRs) with 95% confidence intervals (CIs) were presented with highest tertile of fetuin-A (T3) and T50 (T3) as referent. CRP and T50 were transformed to the common log before entering the regression models. We calculated the sdHRs (95% CIs) of tertiles of fetuin-A and T50 with new CVD events with unadjusted Fine–Gray model. First, the model was minimally adjusted for age and pre-existing CVD (Parsimonious Model 1). These two variables were selected by both forward and backward stepwise methods using either Akaike information criteria or Bayesian information criteria and confirmed by the bootstrap resampling. Second, the model was adjusted for the five major demographic factors (Parsimonious Model 2). Further adjustment was performed for factors related to CKD-MBD (Extended Model 1), anemia in CKD (Extended Model 2), traditional risk factors (Extended Model 3), inflammation and wasting (Extended Model 4), log T50 (Extended Model 5) or fetuin-A (Extended Model 6). The linear trends in risks across tertiles were evaluated by entering the median for each categorical level of exposure. Because the associations of fetuin-A or log T50 with the outcomes were deemed linear, we additionally conducted similar analyses with fetuin-A and log T50 as continuous variables, and sdHRs were expressed per 1-SD lower. As sensitivity analyses, the association of fetuin-A or T50 with new CVD events was evaluated using the Kaplan–Meier curve with log-rank test and Cox proportional hazard regression models. HRs with 95% CIs were presented with the highest tertile (T3) of fetuin-A or T50 as referent. Similar statistical analyses were performed by fetuin-A or log T50 (per 1-SD lower) as continuous variable.

Finally, we focused on deaths after new CVD events. The patients who experienced new CVD events were further followed-up for up to 2.5 years. In addition to the limited numbers of death after CVD events, the death rates were similar between T2 and T3. Therefore, T2 and T3 were combined as T2 + T3 and were compared with T1. Unadjusted associations of fetuin-A and T50 with death after CVD events were examined by Kaplan–Meier method with log-rank test. HRs of fetuin-A and T50 with death after CVD events were further analyzed with unadjusted Cox model and adjusted for age and pre-existing CVD (Parsimonious Model), Parsimonious Model + sex (Extended Model 1), Parsimonious Model + duration of hemodialysis (Extended Model 2), Parsimonious Model + DKD (Extended Model 3), Parsimonious Model + log T50 (Extended Model 4) and Parsimonious Model + fetuin-A (Extended Model 5). These associations were also examined by fetuin-A or log T50 as continuous variable.

These statistical calculations were performed with EZR (version 1.55) developed by Prof. Kanda, Saitama Medical Center, Jichi Medical University, Saitama, Japan, which is graphical interface for R (The R Foundation for Statistical Computing, Vienna, Austria) [33]. More precisely, it is a modified version of R commander designed to add statistical functions frequently used in biostatics. Bootstrap resampling and variable selection procedures were performed using R 4.2.2 software (the R Foundation for Statistical Computing, Vienna, Austria). A *P*-value <.05 by two-sided test was considered statistically significant.

## RESULTS

### Selection and characteristics of study participants

Of the 518 total patients in the DREAM cohort, 513 patients were analyzed for this study (Supplementary data, Fig. S1). In the total patients, serum fetuin-A was normally distributed whereas serum T50 was not. The median (interquartile range) levels of fetuin-A and T50 were 268 (238–304) μg/mL and 176 (140–219) min, respectively. Fetuin-A level was positively associated with T50 (ρ = 0.598, *P* < .001). The baseline characteristics of patients are summarized according to tertiles of fetuin-A and T50 (Table 1).

**Table 1:** Clinical characteristics of 513 patients.

		Fetuin-A				T50			
	Total	T1	T2	T3	*P*	T1	T2	T3	*P*
Number of patients	513	171	170	172		175	168	170	
Fetuin-A (μg/mL)	268 (238–304)	221 (200–238)	267 (258–281)	318 (304–342)	<.001	233 (205–261)	270 (254–300)	305 (274–339)	<.001
T50 (min)	176 (140–219)	138 (118–171)	171 (148–199)	214 (189–248)	<.001	129 (115–142)	177 (165–191)	240 (219–288)	<.001
Age (years)	61 (54–68)	65 (59–72)	62 (55–68)	57 (51–62)	<.001	63 (56–70)	61 (55–67)	60 (53–66)	.017
Male sex (%)	63.2	62	62.9	64.5	.885	63.4	64.3	61.8	.887
Duration of hemodialysis (years)	9.3 (3.8–15.9)	7.7 (3.2–13.0)	8.5 (3.4–14.8)	11.5 (5.4–19.2)	<.001	8.6 (3.5–13.1)	10.5 (3.4–17.0)	9.5 (4.6–17.6)	.237
DKD (%)	21.2	28.1	22.4	13.4	.004	22.9	20.2	20.6	.812
Pre-existing CVD (%)	33.3	42.7	30.6	26.7	.005	40.6	32.7	26.5	.021
Calcium (mg/dL)	9.1 (8.6–9.8)	9.0 (8.5–9.6)	9.1 (8.5–9.8)	9.3 (8.7–9.9)	.010	9.0 (8.4–9.6)	9.1 (8.6–10.0)	9.2 (8.6–9.9)	.012
Phosphate (mg/dL)	5.8 (5.0–6.6)	5.8 (4.9–6.)	5.6 (5.0–6.6)	5.8 (5.1–6.7)	.506	6.1 (5.2–6.8)	5.7 (5.1–6.7)	5.6 (4.7–6.2)	<.001
Intact PTH (pg/mL)	118 (40–214)	125(58–199)	120 (34–226)	113 (37–208)	.478	139 (63–244)	101 (37–182)	112 (35–204)	.036
Use of VDRA (%)	36.3	57 (33.3	40.6	34.9	.341	38.3	39.9	30.6	.163
Hematocrit (%)	30.7 (28.6–32.4)	30.7 (28.4–32.2)	30.5 (28.7–32.5)	31.0 (28.8–32.4)	.626	30.4 (28.3–32.2)	30.7 (28.8–32.3)	31.0 (29.0–32.7)	.140
ESA dose (×1000 U/week)	9.0 (7.5–9.0)	9.0 (9.0–9.0)	9.0 (7.5–9.0)	9.0 (6.0–9.0)	.016	9.0 (9.0–9.0)	9.0 (7.5–9.0)	9.0 (6.0–9.0)	.021
Use of IV iron (%)	57.7	63.2	60.6	49.4	.024	62.9	58.3	51.8	.111
Hypertension (%)	86.2	85.4	88.2	84.9	.626	88	89.9	80.6	.032
Smoker (%)	41.3	38	42.9	43	.559	44	41.7	38.2	.551
Dyslipidemia (%)	49.7	42.1	54.7	52.3	.047	46.3	49.4	53.5	.403
BMI (kg/m^2^)	21.5 (19.6–23.5)	20.8 (19.1–22.4)	21.9 (20.2–23.6)	22.0 (19.8–24.0)	<.001	21.5 (19.6–23.1)	21.2 (19.5–23.3)	21.9 (19.7–23.9)	.445
Serum albumin (g/dL)	3.7 (3.5–3.9)	3.6 (3.4–3.8)	3.7 (3.5–3.9)	3.8 (3.6–4.0)	<.001	3.7 (3.4–3.9)	3.7 (3.6–3.9)	3.7 (3.6–3.9)	.001
CRP (mg/dL)	0.14 (0.05–0.41)	0.25 (0.08–0.86)	0.12 (0.05–0.39)	0.08 (0.04–0.20)	<.001	0.20 (0.07–0.72)	0.14 (0.05–0.32)	0.08 (0.04–0.25)	<.001

Data are expressed as numbers, percentages or median (interquartile range).

*P*-values were by χ^2^ test or by Kruskal–Wallis test.

*P*-values across tertiles.

IV iron, intravenous iron preparation; BMI, body mass index.

### Fetuin-A but not T50 as an independent predictor of new CVD events

**Figure 1:**
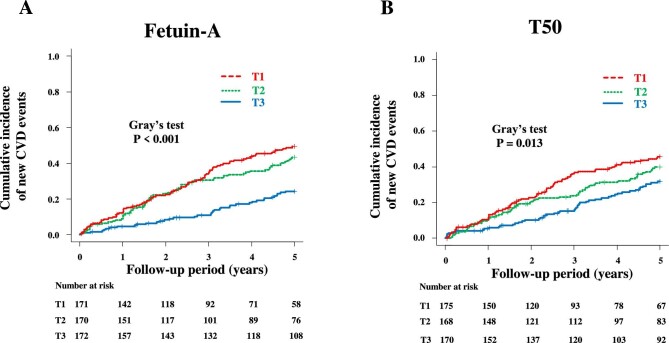
Cumulative incidence of new CVD events in association with fetuin-A (**A**) and T50 (**B**).

We had 190 new CVD events during follow-up period (Supplementary data, Fig. S1). Figure 1 shows cumulative incidence function for new CVD events according to tertiles of fetuin-A and T50 considering the competing risk of death. A lower fetuin-A and a shorter T50 was associated with a higher risk of new CVD events. We examined the unadjusted and adjusted associations of fetuin-A and T50 with new CVD events (Table 2). Compared with T3 as the referent, T1 and T2 of fetuin-A were significantly associated with increased sdHRs in Fine–Gray models unadjusted and adjusted for age and pre-existing CVD (Parsimonious Model 1), the five major demographic factors (Parsimonious Model 2), and additional variables related to CKD-MBD (Extended Model 1), anemia in CKD (Extended Model 2), traditional risk factors (Extended Model 3), inflammation and wasting (Extended Model 4), and log T50 (T2 but not T1) (Extended Model 5). In contrast, T50 was not associated with new CVD events in any adjusted models (Parsimonious Model and Extended Models 1 to 4 and 6).

**Table 2:** Association of fetuin-A or T50 with CVD events by Fine–Gray models.

		Tertiles of fetuin-A				Tertiles of T50			
		T1	T2	T3	Total	T1	T2	T3	Total
Number of cases		81	70	39	190	76	64	50	190
Patient-years		531.7	576.6	688.7	1797.0	556.0	603.2	637.7	1797.0
Crude rate (cases per 1000 patient-years)		152.3	121.4	56.6	105.7	136.7	106.1	78.4	105.7
Model	Adjustment	Subdistribution HR (95% CI)			*P* for trend	Subdistribution HR (95% CI)			*P* for trend
Unadjusted		2.53 (1.74–3.69)***	2.12 (1.44–3.13)***	Ref.	< 0.001	1.70 (1.20–2.42)**	1.38 (0.96–1.98)	Ref.	.002
Parsimonious 1	Age and pre-existing CVD	1.73 (1.15–2.61)**	189 (1.27–2.77)**	Ref.	0.012	1.39 (0.96–2.00)	1.32 (0.92–1.90)	Ref.	.069
Parsimonious 2	Age, sex, duration of hemodialysis, DKD, pre-existing CVD	1.65 (1.09–2.51)*	1.82 (1.23–2.69)**	Ref.	0.027	1.40 (0.97–2.02)	1.36 (0.95–1.96)	Ref.	.063
Extended 1	Parsimonious Model 2 + Ca, P, intact PTH, use of VDRA	1.59 (1.04–2.41)*	1.79 (1.21–2.66)**	Ref.	0.045	1.31 (0.90–1.91)	1.35 (0.94–1.94)	Ref.	.140
Extended 2	Parsimonious Model 2 + Ht, dose of ESA, use of IV iron	1.66 (1.09–2.53)*	1.82 (1.23–2.70) **	Ref.	0.025	1.41 (0.98–2.04)	1.36 (0.95–1.96)	Ref.	.057
Extended 3	Parsimonious Model 2 + Hypertension, smoking, dyslipidemia	1.74 (1.14–2.64)**	1.83 (1.24–2.71)**	Ref.	0.012	1.39 (0.96–2.01)	1.29 (0.90–1.86)	Ref.	.072
Extended 4	Parsimonious Model 2 + BMI, log CRP, albumin	1.57 (1.00–2.44) *	1.77 (1.18–2.64)**	Ref.	0.056	1.36 (0.92–2.00)	1.31 (0.90–1.90)	Ref.	.100
Extended 5	Parsimonious Model 2 + log T50	1.46 (0.91–2.34)	1.70 (1.13–2.57)*	Ref.	0.170				
Extended 6	Parsimonious Model 2 + fetuin-A					1.21 (0.77–1.89)	1.29 (0.88–1.89)	Ref.	.340

Death as a competing risk (Fine–Gray models).

***P < .001, **P < .01, *P < .05.

IV iron, intravenous iron preparation; BMI, body mass index.

Next, fetuin-A or log T50 were entered as continuous variable into Fine–Gray models (Supplementary data, Table S1). Although fetuin-A was significantly associated with CVD events in Parsimonious Model 1, these significant associations disappeared in other adjusted models (Parsimonious Model 2 and Extended Models 1–5). However, when CVD events were divided into atherosclerotic (*n* = 90) and non-atherosclerotic CVD events (*n* = 100), fetuin-A was significantly associated with non-atherosclerotic CVD events in all models except Extended Model 4. On the other hand, no significant association of fetuin-A with atherosclerotic CVD events was found in any model. In contrast, log T50 was not associated with new CVD events regardless of the causes. These findings suggest that fetuin-A was consistently associated with new CVD events, especially non-atherosclerotic CVD events, whereas T50 was not.

### T50 but not fetuin-A as an independent predictor of mortality after CVD events

**Figure 2:**
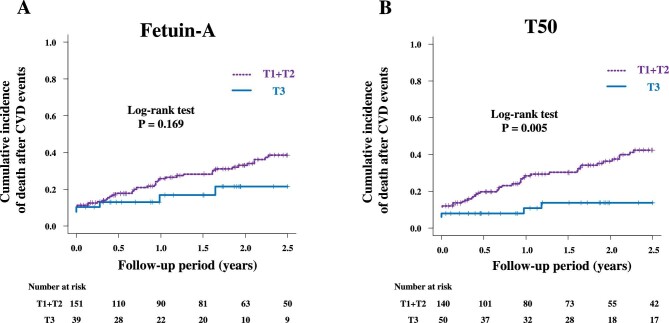
Cumulative incidence of death after new CVD events in association with fetuin-A (**A**) and T50 (**B**).

We focused on deaths after new CVD events. Among the 190 patients who experienced a new CVD event, 59 patients died during the subsequent follow-up for 2.5 years (Supplementary data, Fig. S1). Fetuin-A was not associated with death after CVD events in unadjusted and all adjusted Cox models (Fig. 2A) (Table 3). In contrast, shorter T50 (T1 + T2) were significantly associated with higher mortality after CVD events compared with T3 (Fig. 2B). The inverse association of T50 with mortality after CVD events remained significant after adjustments for any models. Interestingly, the significant association of T50 with mortality after CVD events was independent of fetuin-A (Extended Model 5).

**Table 3:** Association of fetuin-A or T50 with death after CVD events by Cox models.

		Tertiles of fetuin-A				Tertiles of T50			
		T1	T2	T3	Total	T1	T2	T3	Total
Number of cases		28	21	7	56	29	21	6	56
Patients-years		119.0	99.3	51.9	270.2	109.7	85.9	74.6	270.2
Crude rate (cases per 1000 patient-years)		235.3	211.4	134.9	207.2	264.3	244.4	80.4	207.2
		T1 + T2		T3		T1 + T2		T3	
Model	Adjustment	Hazard ratio (95% CI)			*P*	HR (95% CI)			*P*
Unadjusted		1.72 (0.78–3.80)		Ref.	0.180	3.11 (1.33–7.24) **		Ref.	.009
Parsimonious	Age and pre-existing CVD	1.34 (0.60–3.02)		Ref.	0.460	3.31 (1.42–7.74) **		Ref.	.007
Extended 1	Parsimonious Model + sex	1.34 (0.59–3.01)		Ref.	0.460	3.31 (1.42–7.74) **		Ref.	.007
Extended 2	Parsimonious Model + duration of hemodialysis	1.42 (0.63–3.21)		Ref.	0.390	3.20 (1.37–7.50) **		Ref.	.009
Extended 3	Parsimonious Model + DKD	1.34 (0.59–3.01)		Ref.	0.470	3.42 (1.45–8.04) **		Ref.	.007
Extended 4	Parsimonious Model + log 50	0.90 (0.38–2.16)		Ref.	0.810				
Extended 5	Parsimonious Model + fetuin-A					3.24 (1.37–7.65) **		Ref.	.009

**P < .01.

IV iron, intravenous iron preparation; BMI, body mass index.

The preferential association of death after new CVD events with shorter T50 over lower fetuin-A was confirmed in the analyses in which these exposure variables were handled as continuous variables.

### Sensitivity analyses

As sensitivity analyses, we examined the association of fetuin-A or T50 with new CVD events by Cox proportional models in a similar way (Supplementary data, Table S3). Fetuin-A showed significant association with new CVD events in all adjusted models (Parsimonious Models and Extended Models 1–4) except for a model adjusted for T50 (Extended Model 5). In contrast, T50 was not associated with new CVD events in any adjusted models (Parsimonious Models and Extended Models 1–4 and 6). These results by Cox models are almost the same as those by Fine–Gray models (Table 2).

### Fetuin-A, T50 and all-cause mortality

To confirm that our cohort shows the associations of fetuin-A and T50 with all-cause mortality which were reported in previous reports [1, 2, 12, 15], we conducted additional analysis using all-cause mortality as the outcome. During the follow-up period, 104 patients died. Both fetuin-A and T50 were inversely associated with all-cause mortality (Supplementary data, Fig. S2). Both fetuin-A and T50 showed significant association with all-cause mortality in unadjusted and all adjusted Cox models except for a model adjusted for inflammation and wasting (Extended Model 4) in fetuin-A. The significant association of fetuin-A or T50 with all-cause mortality disappeared when adjusted each other (Extended Models 5 and 6; Supplementary data, Table S4).

## DISCUSSION

In this study of hemodialysis patients, we compared the associations of fetuin-A and T50 with two different cardiovascular outcomes, namely the occurrence of new CVD events and the death after the CVD events. Fetuin-A was an independent predictor of new CVD events, especially non-atherosclerotic CVD events, whereas it was not an independent predictor of death after the CVD events. In contrast, T50 was not an independent predictor of new CVD events, whereas it was an independent predictor of death after the CVD events. These results revealed that fetuin-A and T50 are associated with different aspects of cardiovascular risk in this population.

Previous cohort studies examined the prognostic abilities of fetuin-A and T50 in patients with CKD. Ketteler *et al*. were the first to report that a lower serum fetuin-A level was an independent predictor of all-cause death and cardiovascular death using data of patients undergoing hemodialysis [1], although they did not examine the association of fetuin-A with new onset of cardiovascular events or subsequent death. In the study by Smith *et al*. which showed the independent association of T50 with all-cause mortality in 184 patients with CKD stages 3–4, they showed a strong positive correlation between fetuin-A and T50 [12]. However, they did not examine the association of either fetuin-A or T50 with cardiovascular endpoints. Bostom *et al*. analyzed data from a cohort of 685 kidney transplant recipients and reported that both fetuin-A and T50 predicted composite cardiovascular events to a similar extent, although they did not examine the predictive values of fetuin-A and T50 for deaths after CVD events [16]. In this study of hemodialysis patients, we confirmed that fetuin-A and T50 were closely correlated, and that both a lower fetuin-A concentration and a shorter T50 predicted all-cause mortality. Furthermore, we revealed that a lower fetuin-A independently predicted the onset of new CVD events, especially non-atherosclerotic CVD events, whereas T50 did not. In contrast, our results showed a shorter T50 independently predicted deaths after CVD events, whereas fetuin-A did not. Thus, our study is the first that revealed the distinct prognostic abilities of fetuin-A and T50 in predicting the two aspects of cardiovascular outcomes in CKD patients undergoing hemodialysis.

Patients with a lower kidney function have a higher risk of the onset of CVD events [34] and a higher risk of death after CVD events such as myocardial infarction [35]. Patients with kidney failure undergoing hemodialysis have a higher risk of the onset of CVD events and a higher risk of death after CVD events [20, 21]. These elevated risks for the onset of CVD (the first step) and subsequent death (the second step) could synergistically increase the death risk from CVD in these patients [19, 36]. Only a few previous studies reported differential associations of cardiovascular risk factors with the first and second steps of cardiovascular outcomes in patients with CKD. A higher level of non-HDL-C predicted atherosclerotic CVD events such as myocardial and cerebral infarctions but not subsequent death in patients undergoing hemodialysis [22]. Use of VDRAs was associated with lower odds for incident CVD events whereas it was not associated with death after CVD events in hemodialysis patients [23]. Serum level of insulin-like growth factor I (IGF-I) was not independently associated with new CVD events, but a lower IGF-I was an independent predictor of death after CVD events in patients undergoing hemodialysis [24]. Thus, the findings of fetuin-A and T50 in the present study expanded the list of risk factors which predict the onset of CVD events and death after CVD events differently.

The fact that a lower serum fetuin-A predicted the occurrence of new CVD events in this cohort study needs explanations. Since both fetuin-A and T50 are related to toxicity of calcium phosphate crystals, non-mineral actions of fetuin-A may explain the result of this study. First, fetuin-A is profoundly involved in metabolic disorders such as diabetes, dyslipidemia and the metabolic syndrome [8–10, 37]. In addition, hepatic fetuin-A is strongly induced by nutritional factors such as glucose, fructose and fatty acids [38, 39]. Thus, these relationships of fetuin-A with metabolic and nutritional factors may be relevant to its association with the occurrence of new CVD events. Second, lower serum fetuin-A levels may be associated with a higher indoxyl sulfate, one of the major uremic toxins which can strongly produce reactive oxygen species, and potentially cause severe vascular injury. This speculation is based on the report showing that indoxyl sulfate suppressed production of fetuin-A in cultured hepatocytes [40]. Serum indoxyl sulfate and other uremic toxins may be confounding factors that affect the association between fetuin-A and the occurrence of CVD events, but were not adjusted in this analysis. In addition, we found the more preferential association of fetuin-A with non-atherosclerotic CVD events over atherosclerotic CVD events in this study. Fetuin-A-deficient mice showed severe myocardial calcification resulting in impaired cardiac output, diastolic dysfunction and catecholamine resistance [41]. This experimental report supports our clinical findings. What are the mechanisms for the preferential association of T50 with death after CVD events in this cohort of hemodialysis patients? Although fetuin-A is closely associated with T50, T50 is an integrated indicator of calcification stress which is affected not only by fetuin-A but also by calcium, phosphate, magnesium and serum albumin [11]. Thus, these factors may be related to the above-mentioned result of this study. According to a previous cohort study in hemodialysis patients [23], serum levels of calcium and phosphate were significantly predictive of death after CVD events but not predictive of the onset of CVD events. Another cohort study of hemodialysis patients reported that a lower serum albumin was associated not only with incident myocardial infarction and incident cerebral infarction but also death after myocardial infarction and death after cerebral infarction [22]. *In vitro* and *in vivo* studies suggest that magnesium inhibits vascular calcification [42, 43]. Hypomagnesemia was associated with mortality in patients undergoing hemodialysis [44]. In addition, administration of magnesium oxide suppressed coronary artery calcification in CKD patients [45]. Thus, it is possible that these associations of calcium, phosphate, magnesium and albumin, in addition to fetuin-A, are integrated in T50, which may be predictive of the death after CVD events.

This study has certain limitations. First, this was a single-center study in Japan. We cannot generalize our conclusions for other ethnicities and countries. Second, we used single measurements of fetuin-A and T50 at baseline. Variations over time by repeated measures may change the association of fetuin-A or T50 with outcomes. Third, the measured samples were stored for a relatively long time, although we carefully kept them at −80°C without repeated freezing and thawing. Fourth, we did not evaluate serum CPP level directly, although we simultaneously measured both fetuin-A and T50. Fifth, we did not check serum magnesium levels. Sixth, it is possible that statistical power is not enough to detect the association of fetuin-A with mortality after CVD events because of the limited numbers of death after CVD events, although we tried adjustment as carefully as possible. Finally, the results do not necessarily indicate causality because of the observational study design.

In conclusion, we showed the differential associations of fetuin-A and T50 with the occurrence of new CVD events and the death after the new CVD events in a cohort of hemodialysis patients. These results indicate that fetuin-A and T50 are associated with different aspects of cardiovascular risk in this population. Further studies are needed to confirm our observations in a larger cohort and to understand the precise mechanisms behind the observed associations.

## Supplementary Material

sfae042_Supplemental_File

## Data Availability

The data that support the findings of this study are available from the corresponding author (K.M.) upon reasonable request.
