# Supplementary material for: Differential associations of fetuin-A and calcification propensity with cardiovascular events and subsequent mortality in patients undergoing hemodialysis
Source: Clin Kidney J. 2024 Feb 29;17(3):sfae042. doi: 10.1093/ckj/sfae042 (PMC10939447; doi:10.1093/ckj/sfae042)
Supplement: sfae042_Supplemental_File [file sfae042_supplemental_file.pdf]

Supplemental Material (List)

Supplementary Table 1. Association of Fetuin-A or T50 as continuous variable with CVD events by Fine-Gray models

Supplementary Table 2. Association of Fetuin-A or T50 as continuous variable with death after CVD events by Fine-Gray models

Supplementary Table 3. Association of Fetuin-A or T50 with CVD events by Cox models (Sensitivity analysis)

Supplementary Table 4. Association of Fetuin-A or T50 with all-cause mortality by Cox models

Supplementary Figure 1. Flow diagram of patients

Supplementary Figure 2. Cumulative incidence of all-cause mortality in association with fetuin-A (A) and T50 (B)

Supplementary Table 1. Association of Fetuin-A or T50 as continuous variable with CVD events by Fine-Gray models

		Fetuin-A (per 1-SD lower)			Log T50 (per 1-SD lower)		
		CVD	Atherosclerotic CVD	Non-atherosclerotic CVD	CVD	Atherosclerotic CVD	Non-atherosclerotic CVD
Model	Adjustment	Subdistribution HR (95% confidential interval)			Subdistribution HR (95% confidential interval)		
Unadjusted		1.42 (1.23-1.64) ***	1.15 (0.95-1.39)	1.58 (1.28-1.94) ***	1.28 (1.09-1.5) **	1.20 (0.97-1.50)	1.24 (1.00-1.54)
Parsimonious 1	Age and pre-existing CVD	1.19 (1.02-1.39) *	1.00 (0.81-1.22)	1.34 (1.07-1.67) *	1.17 (1.00-1.37)	1.13 (0.91-1.41)	1.15 (0.93-1.42)
Parsimonious 2	Age, sex, duration of hemodialysis, DKD, and pre-existing CVD	1.16 (0.99-1.36)	0.97 (0.79-1.19)	1.33 (1.06-1.68) *	1.17 (1.00-1.37)	1.11 (0.89-1.38)	1.17 (0.95-1.44)
Extended 1	Parsimonious Model 2 + Ca, P, intact PTH, and use of VDRA	1.16 (0.99-1.36)	0.97 (0.78-1.20)	1.32 (1.04-1.68) *	1.13 (0.96-1.33)	1.08 (0.85-1.37)	1.12 (0.90-1.39)
Extended 2	Parsimonious Model 2 + Ht, dose of ESA, use of IV iron	1.17 (1.00-1.37)	1.00 (0.80-1.25)	1.32 (1.06-1.64) *	1.17 (1.00-1.37)	1.14 (0.91-1.43)	1.15 (0.94-1.41)
Extended 3	Parsimonious Model 2 +Hypertension, smoking, dyslipidemia	1.19 (1.01-1.40)	0.99 (0.80-1.22)	1.34 (1.06- 1.7) *	1.17 (1.00-1.38)	1.12 (0.89- 1.40)	1.16 (0.94-1.43)
Extended 4	Parsimonious Model 2 + BMI, log CRP, and albumin	1.14 (0.95-1.37)	0.99 (0.78-1.26)	1.26 (0.96-1.66)	1.16 (0.98-1.38)	1.16 (0.92-1.46)	1.11 (0.89-1.38)
Extended 5	Parsimonious Model 2 + log T50	1.09 (0.90-1.32)	0.87 (0.67-1.13)	1.32 (1.00-1.75) *	—	—	—
Extended 6	Parsimonious Model 2 + fetuin-A	—	—	—	1.11 (0.92-1.35)	1.21 (0.90-1.62)	1.01 (0.80-1.28)

DKD, diabetic kidney disease; CVD, cardiovascular disease; PTH, parathyroid hormone; VDRA, vitamin D receptor activator; IV iron, intravenous iron preparation; BMI, body mass index; CRP, C-reactive protein; HR, hazard ratios; 95% CI, 95% confidential intervals. ***P < 0.001, **P < 0.01, *P < 0.05.

Supplementary Table 2. Association of Fetuin-A or T50 as continuous variable with death after CVD events by Cox models

		Fetuin-A (per 1-SD lower)	Log T50 (per 1-SD lower)
Model	Adjustment	HR (95% confidential interval)	HR (95% confidential interval)
Unadjusted		1.33 (1.01-1.76) *	1.45 (1.1-1.91) **
Parsimonious	Age and pre-existing CVD	1.16 (0.87-1.55)	1.50 (1.13-1.98) **
Extended 1	Parsimonious Model + sex	1.16 (0.87-1.56)	1.51 (1.14-2.00) **
Extended 2	Parsimonious Model + duration of hemodialysis	1.17 (0.87-1.57)	1.49 (1.12-1.97 **
Extended 3	Parsimonious Model + DKD	1.16 (0.87-1.56)	1.51 (1.14-1.99) **
Extended 4	Parsimonious Model + log T50 (per 1-SD)	0.89 (0.63-1.26)	—
Extended 5	Parsimonious Model + fetuin-A (per 1-SD)	—	1.60 (1.13-2.28) **

DKD, diabetic kidney disease; CVD, cardiovascular disease; PTH, parathyroid hormone; VDRA, vitamin D receptor activator; IV iron, intravenous iron preparation; BMI, body mass index; CRP, C-reactive protein; HR, hazard ratios; 95% CI, 95% confidential intervals. ***P < 0.001, **P < 0.01, *P < 0.05.

Supplementary Table 3. Association of Fetuin-A or T50 with CVD events by Cox models (Sensitivity analysis)

		Tertiles of fetuin-A				Tertiles of T50			
		T1	T2	T3	total	T1	T2	T3	total
Number of cases		81	70	39	190	76	64	50	190
Patients-years		531.7	576.6	688.7	1797.0	556.0	603.2	637.7	1797.0
Crude rate (cases per 1,000 patient-year)		152.3	121.4	56.6	105.7	136.7	106.1	78.4	105.7
Model	Adjustment	Hazard ratio (95% confidential interval)			P for trend	Hazard ratio (95% confidential interval)			P for trend
Unadjusted		2.72 (1.86–3.99) ***	2.16 (1.46–3.19) ***	Ref.	< 0.001	1.76 (1.23–2.51) **	1.35 (0.94–1.96)	Ref.	0.002
Parsimonious 1	Age and pre-existing CVD	1.84 (1.23-2.77) **	1.86 (1.25-2.77) **	Ref.	< 0.001	1.40 (0.98-2.01)	1.27 (0.88-1.84)	Ref.	0.006
Parsimonious 2	Age, sex, duration of hemodialysis, DKD, and pre-existing CVD	1.77 (1.18–2.67) **	1.81 (1.21–2.71) **	Ref.	0.009	1.42 (0.99-2.03)	1.31 (0.90-1.90)	Ref.	0.052
Extended 1	Parsimonious Model 2 + Ca, P, intact PTH, and use of VDRA	1.68 (1.11–2.54) *	1.78 (1.19–2.66) **	Ref.	0.024	1.31 (0.90-1.90)	1.28 (0.88-1.87)	Ref.	0.143
Extended 2	Parsimonious Model 2 + Ht, dose of ESA, use of IV iron	1.77 (1.17–2.67) **	1.80 (1.21–2.70) **	Ref.	0.011	1.41 (0.98-2.03)	1.31 (0.90-1.90)	Ref.	0.053
Extended 3	Parsimonious Model 2 +Hypertension, smoking, dyslipidemia	1.92 (1.26–2.92) **	1.84 (1.23–2.74) **	Ref.	0.003	1.40 (0.97-2.02)	1.25 (0.86-1.81)	Ref.	0.062
Extended 4	Parsimonious Model 2 + BMI, log CRP, and albumin	1.56 (1.00–2.41) *	1.72 (1.14–2.59) **	Ref.	0.069	1.28 (0.88-1.86)	1.21 (0.83-1.77)	Ref.	0.181
Extended 5	Parsimonious Model 2 + log T50	1.55 (0.97–2.46)	1.69 (1.12–2.57) *	Ref.	0.096	—	—	—	—
Extended 6	Parsimonious Model 2 + fetuin-A	—	—	—	—	1.08 (0.70-1.68)	1.18 (0.81-1.74)	Ref.	0.623

DKD, diabetic kidney disease; CVD, cardiovascular disease; PTH, parathyroid hormone; VDRA, vitamin D receptor activator; IV iron, intravenous iron preparation; BMI, body mass index; CRP, C-reactive protein; **HR, hazard ratios; 95% CI, 95% confidential intervals.**

***P < 0.001, **P < 0.01, *P < 0.05.

Supplementary Table 4. Association of Fetuin-A or T50 with all-cause mortality by Cox models

		Tertiles of fetuin-A				Tertiles of T50			
		T1	T2	T3	total	T1	T2	T3	total
Number of cases		52	34	18	104	48	34	22	104
Patients-years		675.4	706.1	751.3	2132.9	688.0	714.6	730.3	2132.9
Crude rate (cases per 1,000 patient-year)		77.0	48.1	24.0	48.8	69.8	47.6	30.1	48.8
Model	Adjustment	Hazard ratio (95% confidential interval)			P for trend	Hazard ratio (95% confidential interval)			P for trend
Unadjusted		3.21 (1.88–5.48) ***	2.01 (1.14–3.56) *	Ref.	<0.001	2.31 (1.40–3.83) **	1.58 (0.92–2.70)	Ref.	0.001
Parsimonious 1	Age and pre-existing CVD	1.80 (1.02-3.19) *	1.48 (0.83-2.65)	Ref.	0.044	2.04 (1.23-3.38) **	1.59 (0.93-2.72)	Ref.	0.006
Parsimonious 2	Age, sex, duration of hemodialysis, DKD, and pre-existing CVD	1.88 (1.05–3.36) *	1.66 (0.92–2.98)	Ref.	0.039	2.13 (1.28-3.54) **	1.60 (0.92-2.76)	Ref.	0.004
Extended 1	Parsimonious Model 2 + Ca, P, intact PTH, and use of VDRA	1.94 (1.08–3.47) *	1.62 (0.90–2.92)	Ref.	0.004	2.21 (1.30-3.75) *	1.61 (0.92-2.81)	Ref.	0.003
Extended 2	Parsimonious Model 2 + Ht, dose of ESA, use of IV iron	1.86 (1.04–3.34) *	1.61 (0.89–2.90)	Ref.	0.043	2.06 (1.23-3.43)**	1.60 (0.93-2.76)	Ref.	0.006
Extended 3	Parsimonious Model 2 +Hypertension, smoking, dyslipidemia	1.84 (1.03–3.31) *	1.66 (0.92–3.00)	Ref.	0.050	2.06 (1.23-3.44) **	1.60 (0.92-2.77)	Ref.	0.006
Extended 4	Parsimonious Model 2 + BMI, log CRP, and albumin	1.18 (0.62–2.23)	1.45 (0.80–2.64)	Ref.	0.769	1.73 (1.02-2.96)*	1.57 (0.90-2.74)	Ref.	0.049
Extended 5	Parsimonious Model 2 + log T50	1.34 (0.70–2.56)	1.38 (0.75–2.53)	Ref.	0.462	—	—	—	—
Extended 6	Parsimonious Model 2 + fetuin-A	—	—	—	—	1.63 (0.89-2.97)	1.47 (0.84-2.56)	Ref.	0.120

DKD, diabetic kidney disease; CVD, cardiovascular disease; PTH, parathyroid hormone; VDRA, vitamin D receptor activator; IV iron, intravenous iron preparation; BMI, body mass index; CRP, C-reactive protein. ***P < 0.001, **P < 0.01, *P < 0.05.

Supplementary Figure 1

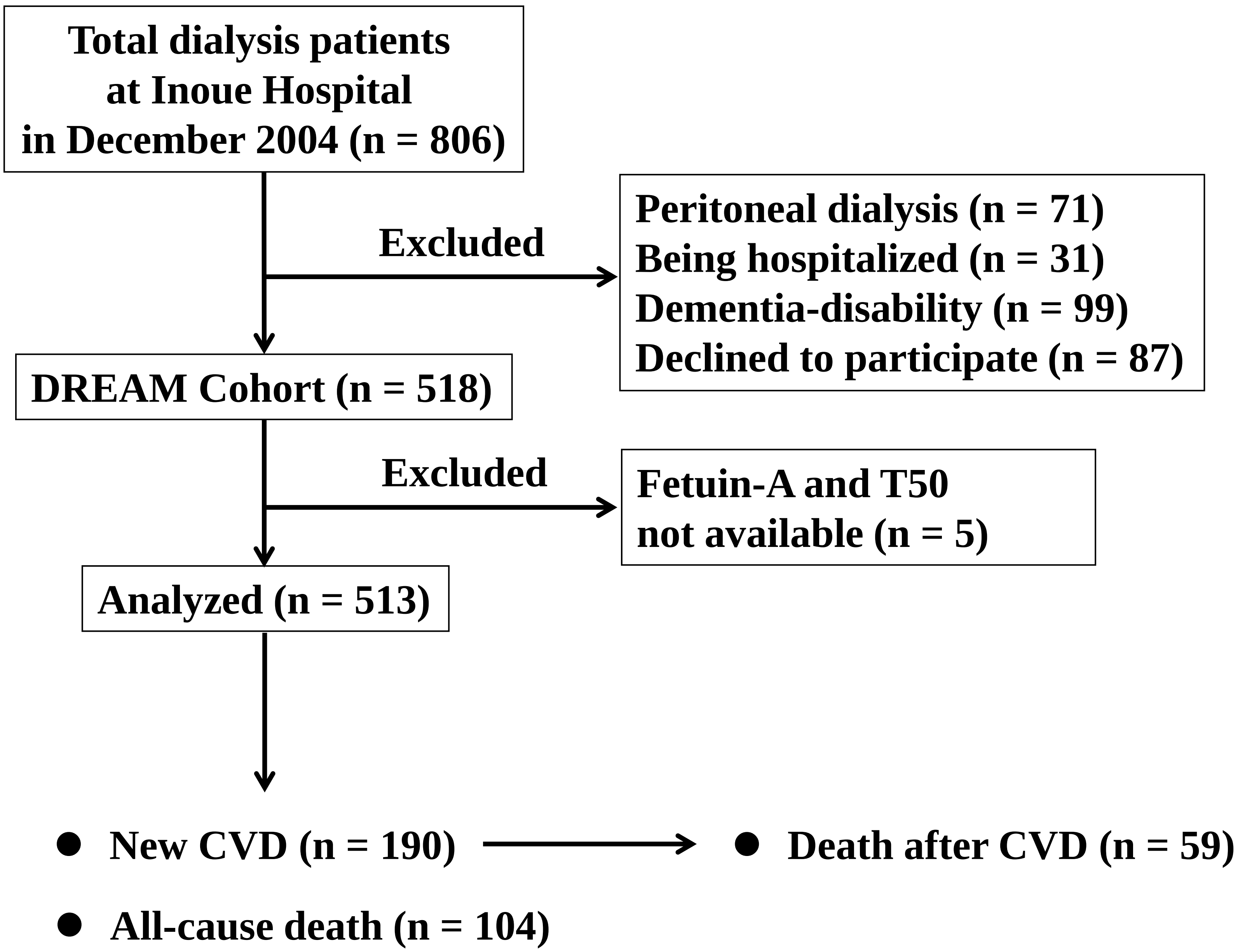

Supplementary Figure 2

A

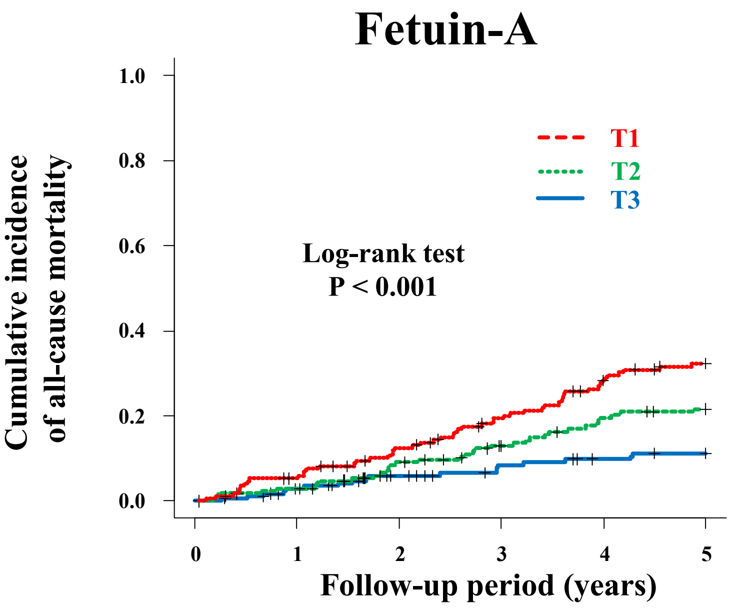

Number at risk

	0	1	2	3	4	5
T1	171	159	143	127	110	102
T2	170	163	145	132	121	117
T3	172	162	152	143	138	137

B

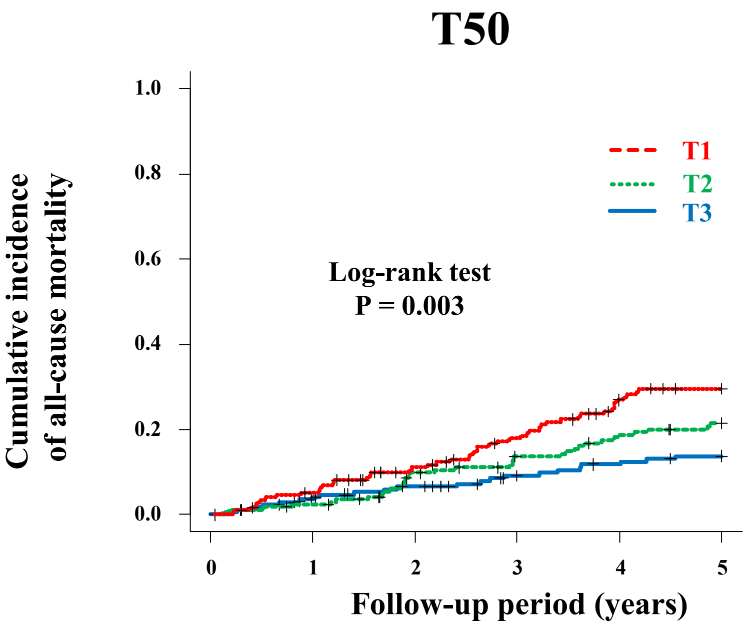

Number at risk

	0	1	2	3	4	5
T1	175	163	145	130	111	108
T2	168	162	144	134	125	120
T3	170	159	151	138	133	130
